# PARADOXICAL ASSOCIATION OF ADIPONECTIN WITH MORTALITY AMONG LONG-LIVED INDIVIDUALS AND THEIR OFFSPRING

**DOI:** 10.1093/geroni/igz038.1791

**Published:** 2019-11-08

**Authors:** Iva Miljkovic, Robert M Boudreau, Ping An, Bharat Thyagarajan, Kaare Christensen, Nicole Schupf, Alexander Kulminski, Joseph M Zmuda

**Affiliations:** 1 University of Pittsburgh, Pittsburgh, Pennsylvania, United States; 2 St. Louis, Washington University, Missouri, United States; 3 Department of Laboratory Medicine and Pathology University of Minnesota; Minneapolis, Minnesota, United States; 4 Danish Aging Research Center University of Southern Denmark, Odense C, Denmark; 5 Columbia University, Columbia University, United States; 6 Duke University, Durham, North Carolina, United States; 7 Department of Epidemiology, University of Pittsburgh; Pittsburgh, Pennsylvania, United States

## Abstract

Adiponectin is an adipokine with well-recognized roles as an insulin-sensitizing, anti-inflammatory, and cardioprotective factor. Despite these biological properties, higher circulating adiponectin levels have recently emerged as a potential risk factor for mortality among older adults. We evaluated the relationship between adiponectin and all-cause mortality further using data collected on 3525 men and women who were participants in the Long Life Family Study (LLFS), a multicenter cohort study of two-generation families with a clustering of healthy aging and exceptional survival. Fasting serum adiponectin levels were measured at study entry. Date of death was abstracted from death certificates and proxy. Cox proportional hazards models were used to estimate the risk of mortality per one SD increase in adiponectin. We adjusted for age, sex, field center, education, lifestyle factors, BMI, comorbid conditions (diabetes, hypertension, lung diseases, heart disease, stroke, and cancer) and intra-familial correlation structure. The mean age of participants at study entry was 71.7 ± 16.3 years (range, 32-110 years; 44.4% male). A total of 1147 deaths occurred during an average 11 years of follow-up (25295 person-years). Higher serum adiponectin was associated with increased mortality even after adjusting for several potential confounding factors (Hazard ratio [95% CI]: 1.19 [1.12, 1.27]; P<0.0001). Consistent with prior studies and contrary to its beneficial metabolic properties, higher adiponectin level was associated with greater all-cause mortality among long-lived individuals and their offspring. Further studies are needed to assess the potential mechanisms underlying this paradoxical association.

